# Few-shot EEG sleep staging based on transductive prototype optimization network

**DOI:** 10.3389/fninf.2023.1297874

**Published:** 2023-12-06

**Authors:** Jingcong Li, Chaohuang Wu, Jiahui Pan, Fei Wang

**Affiliations:** School of Software, South China Normal University, Guangzhou, China

**Keywords:** meta-learning, few-shot, transductive prototype optimization, sleep stage, EEG

## Abstract

Electroencephalography (EEG) is a commonly used technology for monitoring brain activities and diagnosing sleep disorders. Clinically, doctors need to manually stage sleep based on EEG signals, which is a time-consuming and laborious task. In this study, we propose a few-shot EEG sleep staging termed transductive prototype optimization network (TPON) method, which aims to improve the performance of EEG sleep staging. Compared with traditional deep learning methods, TPON uses a meta-learning algorithm, which generalizes the classifier to new classes that are not visible in the training set, and only have a few examples for each new class. We learn the prototypes of existing objects through meta-training, and capture the sleep features of new objects through the “learn to learn” method of meta-learning. The prototype distribution of the class is optimized and captured by using support set and unlabeled high confidence samples to increase the authenticity of the prototype. Compared with traditional prototype networks, TPON can effectively solve too few samples in few-shot learning and improve the matching degree of prototypes in prototype network. The experimental results on the public SleepEDF-2013 dataset show that the proposed algorithm outperform than most advanced algorithms in the overall performance. In addition, we experimentally demonstrate the feasibility of cross-channel recognition, which indicates that there are many similar sleep EEG features between different channels. In future research, we can further explore the common features among different channels and investigate the combination of universal features in sleep EEG. Overall, our method achieves high accuracy in sleep stage classification, demonstrating the effectiveness of this approach and its potential applications in other medical fields.

## 1 Introduction

Electroencephalogram (EEG) is a method for detecting brain signals (Ismail et al., 2016). It uses tiny electrodes attached to the scalp to detect electrical activity in the brain. The EEG signals generated by brain thinking activity can be analyzed and processed by corresponding analysis algorithms, and then converted into corresponding commands to control computers or electronic devices (Hramov et al., 2021).

In recent years, non-invasive brain–computer interfaces (BCI) have achieved significant results in the acquisition of EEG signals (Galán et al., 2008). BCI have been applied in many fields such as sleep signal acquisition, disease diagnosis, emotion analysis, and robot control, which have broad application prospects (Allison et al., 2007).

The application of EEG in monitoring sleep quality is also essential (Sadeh, 2015). Sleep staging can monitor the quality of each sleep segment and determine a person's sleep quality (Carskadon et al., 2005). In the auxiliary diagnosis of some sleep-related diseases such as epilepsy and sleep apnea, sleep staging plays an important role in the diagnosis of the disease (Samy et al., 2013) and can help improve our analysis of these related diseases. The identification of sleep stages is crucial for the diagnosis of sleep disorders, among which obstructive sleep apnea (OSA) is one of the most common diseases (Korkalainen et al., 2019). Traditional manual sleep staging using EEG signals is time-consuming and laborious since it requires analyzing sleep stages from the entire night's sleep signal.

In recent years, a research method for EEG sleep staging using deep learning algorithms has been proposed. Deep learning is a new research direction in the field of machine learning (Arel et al., 2010). It is introduced into machine learning to make it closer to its original goal of artificial intelligence (AI) (Arrieta et al., 2020). Deep learning is a complex machine learning algorithm that learns the internal rules and representation levels of sample data. The information obtained during the learning process is very helpful for interpreting data such as text, images, and sound (LeCun et al., 2015; Tsinalis et al., 2016). The ultimate goal is to enable machines to analyze and learn like humans and recognize data such as text, images, and sound. Deep learning is a powerful tool in the processing of EEG signals and has shown excellent performance in speech and image recognition (Amin et al., 2019; Sun et al., 2019). Traditional EEG sleep staging algorithms include deep learning algorithms or end-to-end trained deep learning algorithms, including convolutional neural network (CNN) or recurrent neural network (RNN) algorithms (Dong et al., 2017; Chambon et al., 2018; Phan et al., 2018; Perslev et al., 2019; Qu et al., 2020), involving state-of-the-art sleep staging networks such as DeepSleepNet (Supratak et al., 2017) and SeqSleepNet (Phan et al., 2019).

The traditional approach to deep learning research involves obtaining a large dataset for a specific task and training a model from scratch using that dataset (Dietterich, 1997). Although deep learning models can achieve high accuracy, the training time and computational cost of this method are significant due to the requirement for large amounts of data (Alzubaidi et al., 2021). In the case of unfamiliar subjects, using deep learning algorithms would require re-calculating, which would consume a considerable amount of computational time and resources. Furthermore, data from different cohorts may come from varying sources due to variations in the number and location of EEG channels, sampling frequency, experimental paradigms, and subject variability, making models trained on one cohort not directly applicable to another, limiting their applicability in clinical settings (Boostani et al., 2017; Andreotti et al., 2018).

Meta-learning, also referred to as learning to learn, involves a systematic examination of the performance of various machine learning methodologies across a diverse spectrum of learning tasks. This process enables the acquisition of knowledge from the amassed meta-data, allowing for significantly accelerated learning of novel tasks beyond conventional capabilities (Vanschoren, 2019). This not only expedites and enhances the development of machine learning workflows and neural network architectures but also facilitates the replacement of manually engineered algorithms with innovative data-driven approaches. Yaohui Zhu proposed a multi-attention meta-learning (MattML) method for few-shot finegrained image recognition (FSFGIR) (Zhu et al., 2020). Instead of using only base learner for general feature learning, the proposed meta-learning method uses attention mechanisms of the base learner and task learner to capture discriminative parts of images.

Meta-learning algorithms can enable cross-subject EEG sleep staging, greatly reducing the training time required for sleep staging. Nannapas proposed a meta-learning MAML-based method, MetaSleepLearner, for sleep staging EEG signals (Finn et al., 2017; Banluesombatkul et al., 2020). They introduced a transfer learning framework based on model-agnostic meta-learning (MAML) to transfer acquired sleep stage knowledge from a large dataset to new individual subjects. The accuracy achieved on the Fpz-Cz validation channel was 72.1% and the MF1 score was 64.8, demonstrating the feasibility of cross-subject EEG sleep staging. Shi et al. (2023) used meta-transfer learning, proposed MTSL, further improved the feature extracter based on the meta-learning framework, and introduced the idea of transfer learning to improve the performance of sleep staging in small sample scenarios through a new meta-transfer framework, and achieved 79.8% ACC on sleep-EDF. In MetaSleepLearner, the experiment uses too many training sets for hybrid training and requires fine-tuning on unused subject data, so the complexity of the experiment does not favor a specific implementation. In MTSL, a multi-stream parallel CNN network is used to extract EEG features from each of the three scales, and finally, the multi-scale features are fused through feature splitting to obtain the final EEG feature representation. Since the network features are too complex, the running time and computational consumption are too large and difficult to implement.

In this study, we propose a few-shot EEG sleep staging based on transductive prototype optimization network (TPON) method to improve the accuracy of cross-subject EEG sleep staging. The aim is to improve the performance of cross-subject EEG sleep staging and also to achieve innovative cross-channel recognition of sleep with good performance. Our experiments are carried out with 20 subjects in the Sleep-EDF dataset, which has a small amount of data and moderate network complexity. We use the prototype network model in meta-learning. Compared with traditional machine learning and other meta-learning methods, our experiment has shorter training time and higher accuracy improvement. Our experiment is based on the prototype network method of meta-learning proposed by Snell et al. (2017). To improve it, we utilize the Transductive Distribution Optimization (TDO) algorithm proposed by Liu et al. (2023). Our experiment is conducted on the Fpz-Cz and Pz-Oz channels and uses the AASM (Berry et al., 2012) scoring standard, which classifies sleep into five stages.

The main contributions of this study are as follows:

- We propose a few-shot EEG sleep staging based on transductive prototype optimization network (TPON) method to improve the performance of cross-subject EEG sleep staging.
- By using few-shot learning and TPON method, we effectively alleviated the problem of too few samples in sleep staging and improved the generalization ability to new subjects.
- In the five-way 15-shot scenario, the cross-subject sleep staging accuracy of TPON can be improved to 87.1%, MF1 to 81.7, and the cross-channel sleep staging can also achieve an accuracy of 82.4%. Additionally, we first experiment and discuss the feasibility of cross-channel sleep staging recognition.

## 2 Proposed method

In this study, we propose a few-shot EEG sleep staging based on transductive prototype optimization network (TPON) method to improve the accuracy of cross-subject EEG sleep staging. Our experiments are based on the prototypical network approach of meta-learning, where prototypes are used in combination with high confidence unlabeled samples to achieve subject transfer.

### 2.1 Overall framework of TPON

**Figure 1:**
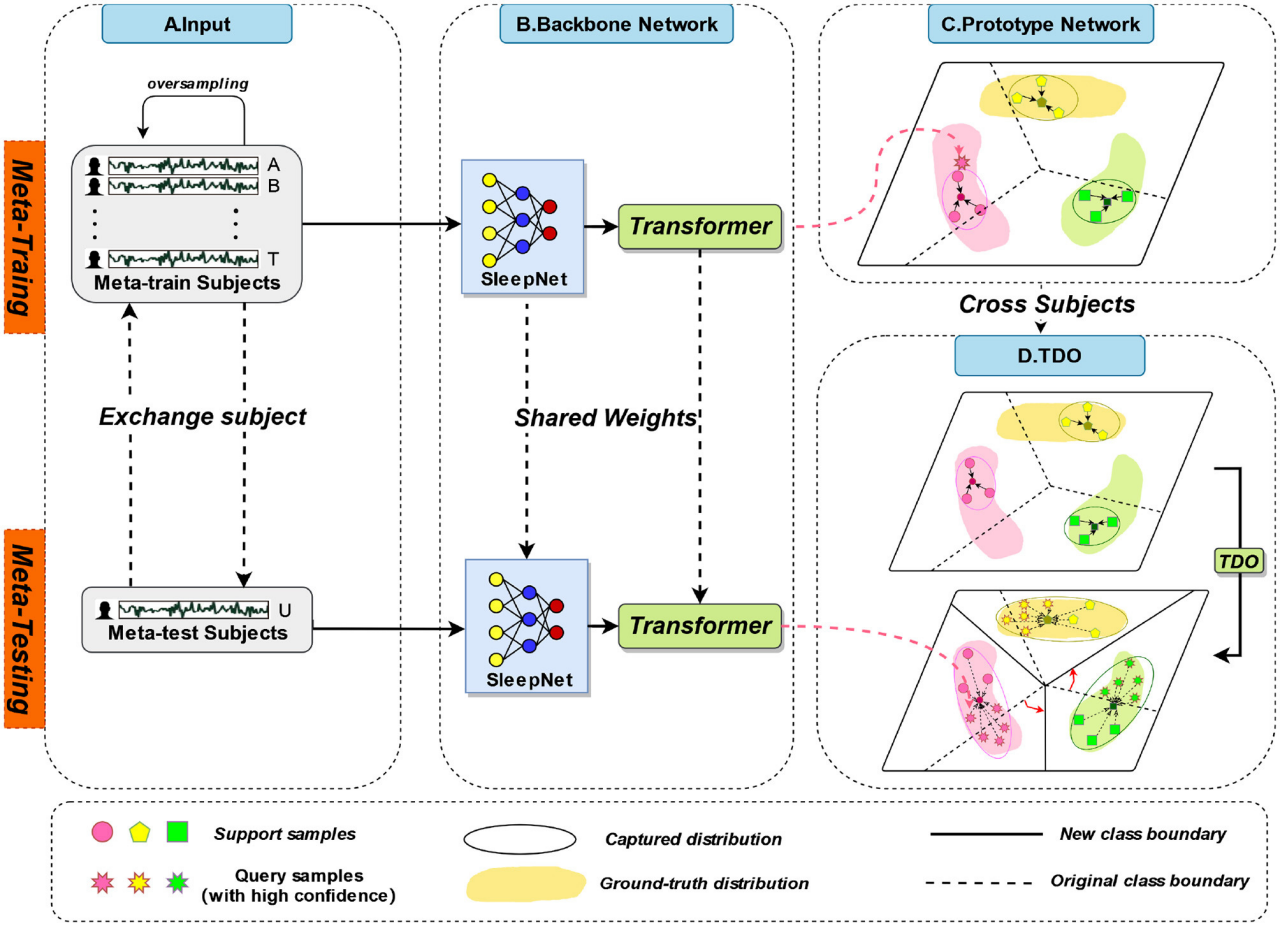
The overall framework of TPON.

The overall framework of TPON is depicted in Figure 1. Different subjects will be used for both meta-training and meta-testing, which is shown at stage A in Figure 1, 19 of them for meta-training and the remaining one for meta-testing. In the meta-training phase, we combine the sleep data of 19 meta-training subjects. Each participant had two nights of data. In the meta-testing phase, data from two nights of one meta-testing subject are combined. We also cross-tested 20 times to obtain average results for all subjects. During meta-testing and meta-training, the sleep network shares the weight values. In the meta-training stage, prototypes of five sleep cycles are obtained through 50 experiments and randomly averaged sampling. Then, during our meta-testing phase, unlike meta training, due to the few-shot size of the meta-testing set.

Phase B in Figure 1 is the backbone network we used, including SleepNet combined with Transformer. The C and D stages in Figure 1 are the improved prototype network and transformation distribution optimization (TDO) methods we used, respectively. The mentioned three phases are discussed in detail in the following sections.

We utilizing the various distance metric functions, including the Cosine distance formula, the Manhattan distance formula, the Euclidean distance formula, and the Chebyshev distance formula. By comparison, we then obtain the highest accuracy among the four. We identify the class to which a test EEG data segment belongs based on its proximity to the prototype. We then compute the average accuracy, precision, loss, and F score for each of the five categories.

### 2.2 Prototypical networks

**Figure 2:**
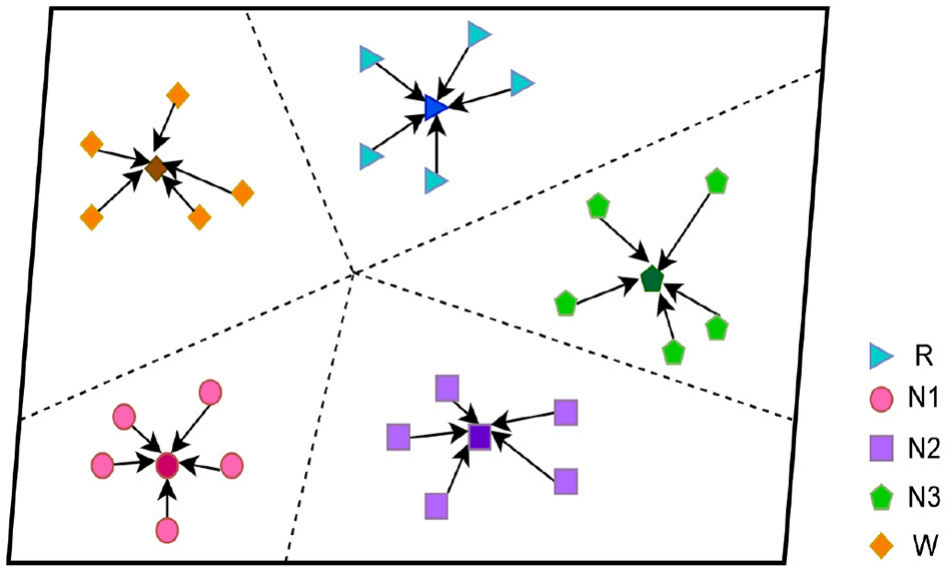
Prototypical network.

In this study, we focus on the prototypical network approach, which is shown in Figure 2 and stage C in Figure 1. The method is based on the classification of sleep EEG signals. We have developed our prototypical network model, which maps sleep EEG signals to embedding vectors and uses their clustering for classification (Hori et al., 2001). The novel feature of our model is that it constructs a richer embedding space through a learned prototypical network related to EEG sleep, such that EEG signals can be projected there. In the Figure 2, we show the five-way five-shot during the five periods of sleep. They are clustered under a distance metric of orientation and class relevance, which is then used for classification (Schultz and Joachims, 2003; Chen et al., 2009).

In few-shot learning (Sung et al., 2018; Wang et al., 2020), if our task is an N-way K-shot, then the support set S, with K-labeled samples, can be expressed as follows:


(1) $$\text{S=}\{({\text{X}}_{1},{\text{Y}}_{1}),({\text{X}}_{2},{\text{Y}}_{2}),\ldots ,({\text{X}}_{\text{N}},{\text{Y}}_{\text{N}})\}$$

Each prototype is an average vector of embedded support points belonging to its class. To better represent the features of each class, the average value of the features of each class is computed by the backbone network F, which is called the prototype *C*_q_. Under the K-shot dimension, *x*_i_∈{1, …, *K*} is the eigenvector of class *i*, and *i* is any one of the *N* classes, *y*_i_∈{1, …, *K*} are the labels of the corresponding category. Then, *S*_q_ respectively represent the support set of class *q*. $\left|S_{q}\right|$ is expressed as the absolute value of *S*_q_. The calculation formula is as follows:


(2) $$\begin{array}{l} C_{q}=\frac{1}{\left|S_{q}\right|}\underset{\left(x_{i},y_{i}\right)\in S_{q}}{\sum }F\left(x_{i}\right), \end{array}$$

Based on the distance from the embedding space to the prototype, the distance metric function d is given, the prototypical network generates a distribution over classes for the query point x using a softmax activation function, which is computed as follows :


(3) $$P(y=q|x)=\frac{\exp(-d(F(x_{i}),C_{q}))}{\sum_{q^{\prime }}\exp(-d(F(x_{i}),C_{q^{\prime }}))},$$

Specifically, *P*(*y* = *q*|*x*) means that the query sample *x* is compared to all $C_{q^{\prime }}$ prototypes, classifying *x* as a probability value of class *q*.

A common prototypical network consists of an backbone network that maps sleep EEG signals to embedding vectors. One batch contains a subset of the available training EEG signals. EEG data from each class is randomly split into support and query sets. The embedding of the support set is used to define the class prototype, i.e., the prototype embedding vector of the class. By using a metric function to measure the distance between the query set and the prototype, the query set is classified.

### 2.3 Distance metric

For prototypical networks and matching network, any measurement function is allowed. In our experiment, *d*_Cos_ means Cosine distance, *d*_Man_ means Manhattan distance, *d*_Euc_ means Euclidean distance, and *d*_Che_ means Chebyshev distance, they were used as comparisons. We obtain the cosine distance as our best matching and most accurate measurement function.

If there is a query sample *Z*_f_, its high-dimensional spatial characteristics can be expressed *F*(*Z*_f_), and *n* represents the dimension of the vector. Therefore, the distance function can be used to obtain the distance between the high-dimensional vector *F*(*Z*_f_) of our query sample and the prototype *C*_q_. The distance calculation formula is as follows:


(4) $$\begin{array}{l} d_{\text{Cos}}\left(F\left(Z_{f}\right),C_{q}\right)=-\cos\theta =-\frac{F\left(Z_{f}\right)\cdot C_{q}}{\left||F\left(Z_{f}\right)||||C_{q}|\right|}, \end{array}$$

(5) $${\text{d}}_{\text{Man}}\left(\text{F}\left({\text{Z}}_{\text{f}}\right),{\text{C}}_{\text{q}}\right)\text{=}\int_{k=1}^{n}\left|\text{F}\left({\text{Z}}_{\text{f},\text{k}}\right){-\text{C}}_{\text{q},\text{k}}\right|,$$

(6) $${\text{d}}_{\text{Euc}}\left(\text{F}\left({\text{Z}}_{\text{f}}\right),{\text{C}}_{\text{q}}\right)\text{=}({\int_{k=1}^{n}{\left|\text{F}\left({\text{Z}}_{\text{f},\text{k}}\right){-\text{C}}_{\text{q},\text{k}}\right|}^{2})}^{\frac{1}{2}},$$

(7) $${\text{d}}_{\text{Che}}\left(\text{F}\left({\text{Z}}_{\text{f}}\right),{\text{C}}_{\text{q}}\right)\text{=}\underset{\text{f}}{\text{MAX}}\left|\text{F}\left({\text{Z}}_{\text{f}}\right){-\text{C}}_{\text{q}}\right|,$$

After the Cosine distance between the query sample and the prototype *C*_q_ is obtained, the negative value of the Cosine distance between the query sample *Z*_f_ and the prototype *C*_q_is formed into a probability distribution on the class through the softmax function. The calculation formula is as follows:


(8) $$P(y_{n}|Z_{f})=\frac{\exp(-\text{dist}(F(Z_{f}),C_{q}))}{\sum_{n=1}^{q}\exp(-\text{dist}(F(Z_{f}),C_{q}^{\prime }))},$$

where *C*_*n*_(n = 1, 2, …, Q) is the prototype of class *n* and dist() can represent four different distance measurement functions.

At the same time, to have a good evaluation index during model training, the experiment uses cross entropy as loss function to train and then minimizes the loss function. The calculation formula is as follows:


(9) $$\begin{array}{l} \text{Loss}=-\frac{1}{n}\overset{n}{\underset{i=1}{\sum }}\overset{q}{\underset{n=1}{\sum }}y_{in}\times \log\left(p\left(y_{n}|Z_{f}\right)\right), \end{array}$$

where *n* is the number of query samples and *y*_i_ is the actual label of the sample.

### 2.4 Transductive distribution optimization

Due to the small number of subjects per sleep period in the sleep data SleepEDF-2013, the problem is that the selected sleep segments do not accurately describe our actual prototypes. To solve this problem, we used the TDO method based on the original prototype network.

**Figure 3:**
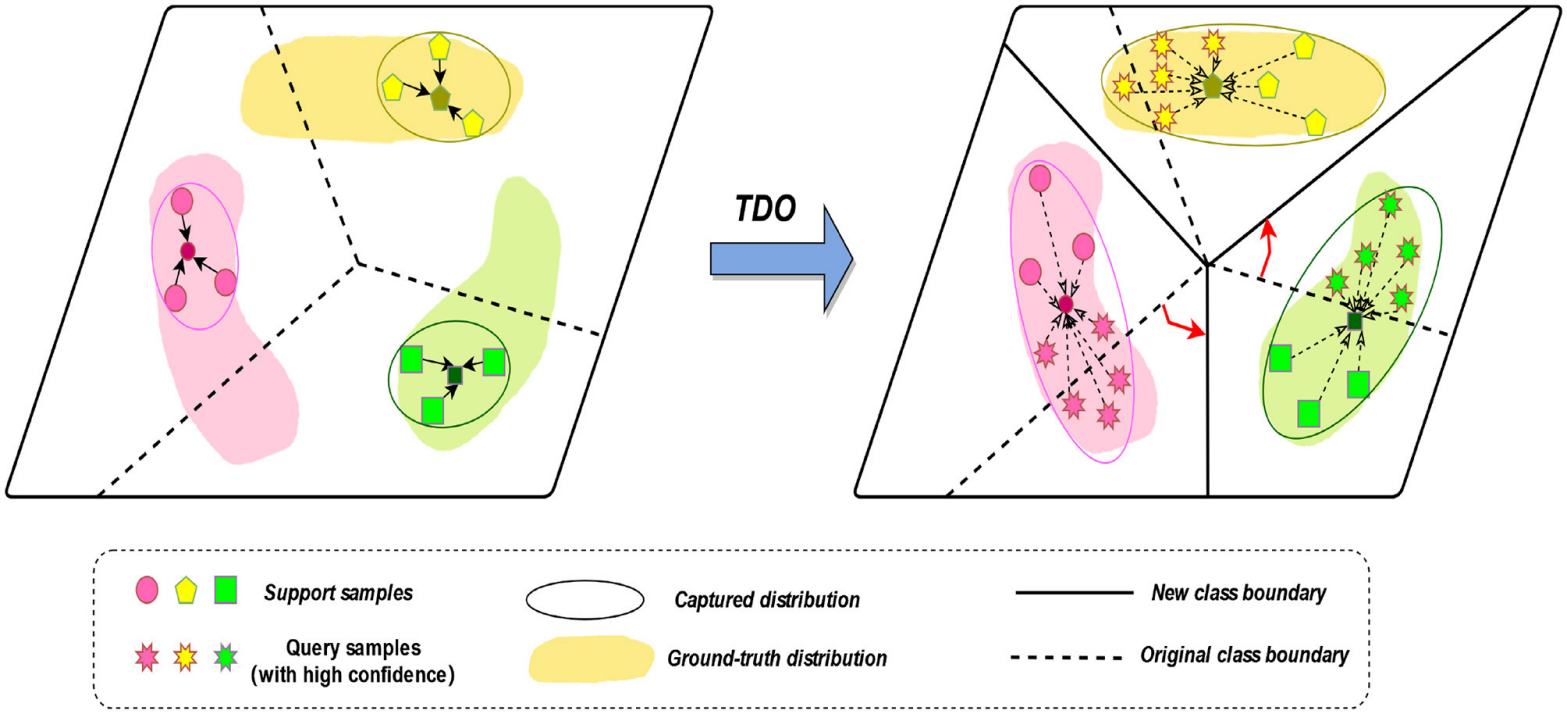
The transductive distribution optimization (TDO) method.

We propose to use the prototype network approach of TDO to capture the features of new classes. We first used the original method, using labeled samples from the five sleep epochs as a support set, to obtain prototypes of the five sleep epochs. However, due to the small number of samples in the learning process, it is not possible to accurately obtain the true distribution of each class. Therefore, we introduce TDO, which combines support set and high-confidence unlabeled sample query set to improve the matching degree of prototypes in prototype network, which is depicted in Figure 3 and stage D in Figure 1. Algorithm 1 summarizes the prediction process of our proposed method.

**Algorithm 1:**
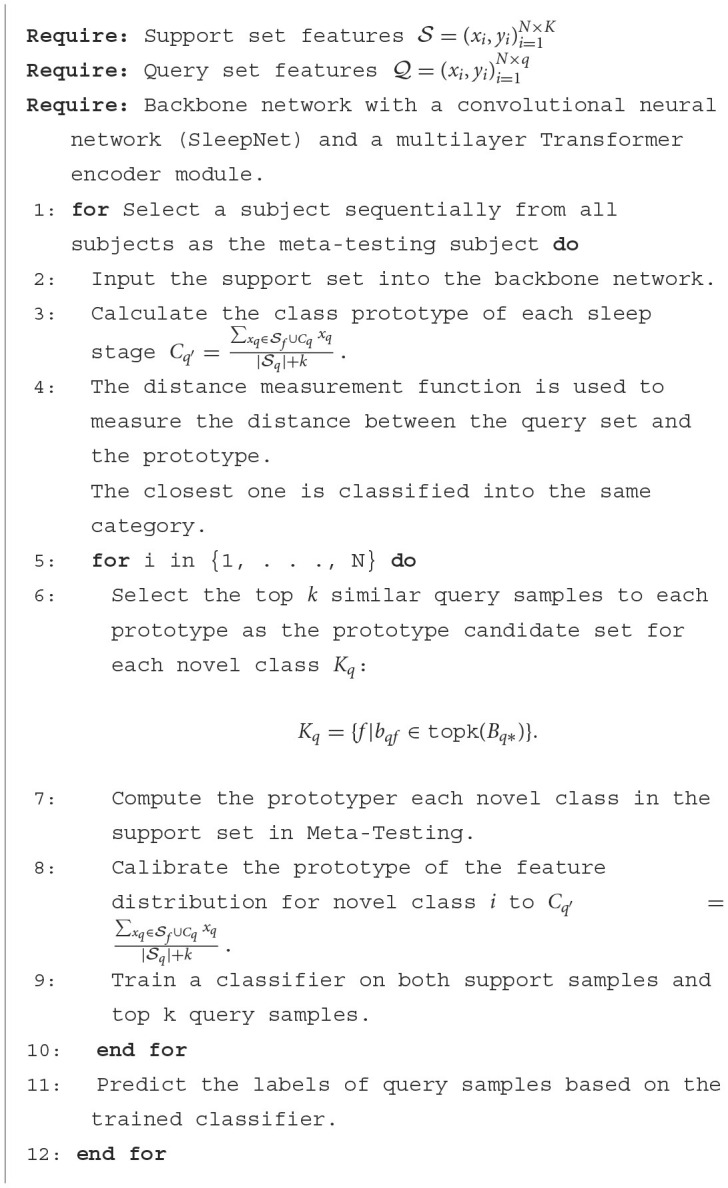
Transductive prototype optimization network (TPON) algorithm.

The main steps are illustrated in the figure above, which include the following three parts:

#### 2.4.1 Generate an original class boundary

In the first stage, we generate an original class boundary using the backbone network and the labeled data. The feature extraction prototype network extracts an original prototype for a five-way N-shot task by using the backbone network, generating an original class boundary, where all the support set samples come from labeled data. For an N-way K-shot task, to find out the similarity scores between all query samples and the support classes, we use the sample-to-class metric measure to get the relational matrix *R*^(N × q) × N^ of similarity probability scores.

#### 2.4.2 Generate a new class boundary

The class distribution is optimized by using a robust feature extractor to capture the feature distribution of each class. It is based on the original support set and some highly confident unlabeled query samples to obtain a ground-truth prototype of the transformed distribution. New class boundaries are generated by combining the original support set and some highly confident unlabeled query samples. The goal is to generate a new classifier that predicts the labels of all remaining query samples.

Then, we obtain a similarity probability score matrix *B*∈*R*^(N × q) × N^ between each prototype *C*_q_ and all query samples *Z*_f_. For each class prototype *C*_q_, we select the top k query samples with the highest similarity probability score as the prototype class candidate set:


(10) $$\begin{array}{l} K_{q}=\{f|b_{qf}\in topk(B_{q*})\},\\ C_{q}=\{x_{q}|x_{q}\in Q,q\in K_{q}\}, \end{array}$$

topk(·) is an operator to select the top *k* elements from each row of the matrix *B*, *k* is a hyperparameter that denotes the number of samples in the prototype class candidate set for each class, and *Q* denotes the query set after Tukey's transformation. Ki stores the index of the *k* most similar query samples of class *i* and Ci stores the samples corresponding to Ki.

#### 2.4.3 Generate a new classifier

A new classifier is generated by combining the original support set and some highly confident unlabeled query samples to generate new class boundaries. With the goal of predicting the labels of all remaining query samples, the mean of the feature distribution for each class is then computed using the support set and the candidate set of prototype classes:


(11) $$C_{q}^{\prime }=\frac{\sum_{x_{q}\in S_{f}\cup C_{q}}x_{q}}{|S_{q}|+k},$$

where $\left|S_{q}\right|$ denotes the number of samples in *S*_*q*_ and *k* denotes the number of samples in *C*_q_. This significantly removes the distribution bias caused by the category mismatch. Our method does not introduce any additional rational parameters and can be paired with most classification models and feature extractors. The introduction of this approach does not add a significant amount of computation, but it can greatly improve the classification accuracy and achieve significant learning results.

## 3 Dataset and experimental setup

In this section, we present the details of our experiments on the proposed method, including dataset and experimental setup. For our experiments, the hardware and software configurations used in our experiments are based on a platform with an Nvidia RTX 3090Ti, Ubuntu 16.04, and PyTorch 1.9.0.

### 3.1 Datasets

This section describes the use and preprocessing of the experimental data. The experiment used the benchmark sleep data disclosed by PhysioNet Sleep-EDF, which included 20 healthy subjects (26–35 years old), including 10 healthy men and 10 healthy women. The polysomnography (PSG) recording time of each person is about 20 h. This dataset includes sleep EEG of healthy subjects' SC. The * PSG.edf as the suffix contains EEG (from Fpz-Cz, Pz-Oz electrode positions) and the * Hypnogram.edf files contain the notes of sleep mode corresponding to PSG. The sampling rate was 100 Hz for all EEG.

Sleep experts manually divide these records into eight categories (W, N1, N2, N3, N4, REM, MOVEMENT, and UNKNOWN). These modes (hypnograph) include sleep stages W, N1, N2, N3, N4, REM, M (body movement time), and “?” (unknown time). This PSG is segmented into 30-s epochs, which are then be classifified into different sleep stages by the experts according to sleep manuals such as the Rechtschaffen and Kales (R&K).

We combined the N3 and N4 phases into a single phase N3 to maintain the AASM standard (Berry et al., 2012). At the beginning and end of each recording, there is a long period of W-phase in which the subject is not sleeping, which we cut off. We only include 30 min before and after the sleep time, and delete M (body movement time) and “?” (unknown time). The notes during EEG sleep have been given separately in the hypnographic files available in the database. Sleep notes are provided every 30 s in each EEG signal to note which sleep stage it belongs to. We divided the sleep EEG of 20 healthy subjects into meta-training subjects and meta-testing subjects. One of the 20 subjects was used as a meta-testing subject, and the remaining 19 subjects were tried as our meta-training subjects, so we can do a 20-fold cross check. We take a time window of 30 s to intercept the sleep samples. Sleep data from two nights for each subject were fused into one subject.

### 3.2 Data enhancement

There is a problem of imbalanced categories in the dataset of SleepEDF-2013. The number of a certain category of training is too small during the meta-training. To solve this problem, we adopted the method of oversampling in the meta-training dataset and kept the number of meta-training data consistent during the five sleep periods by randomly copying the original category of EEG. Let the backbone network learn the category information in an efficient and balanced way without the problem of class imbalance.

### 3.3 Backbone network

**Figure 4:**
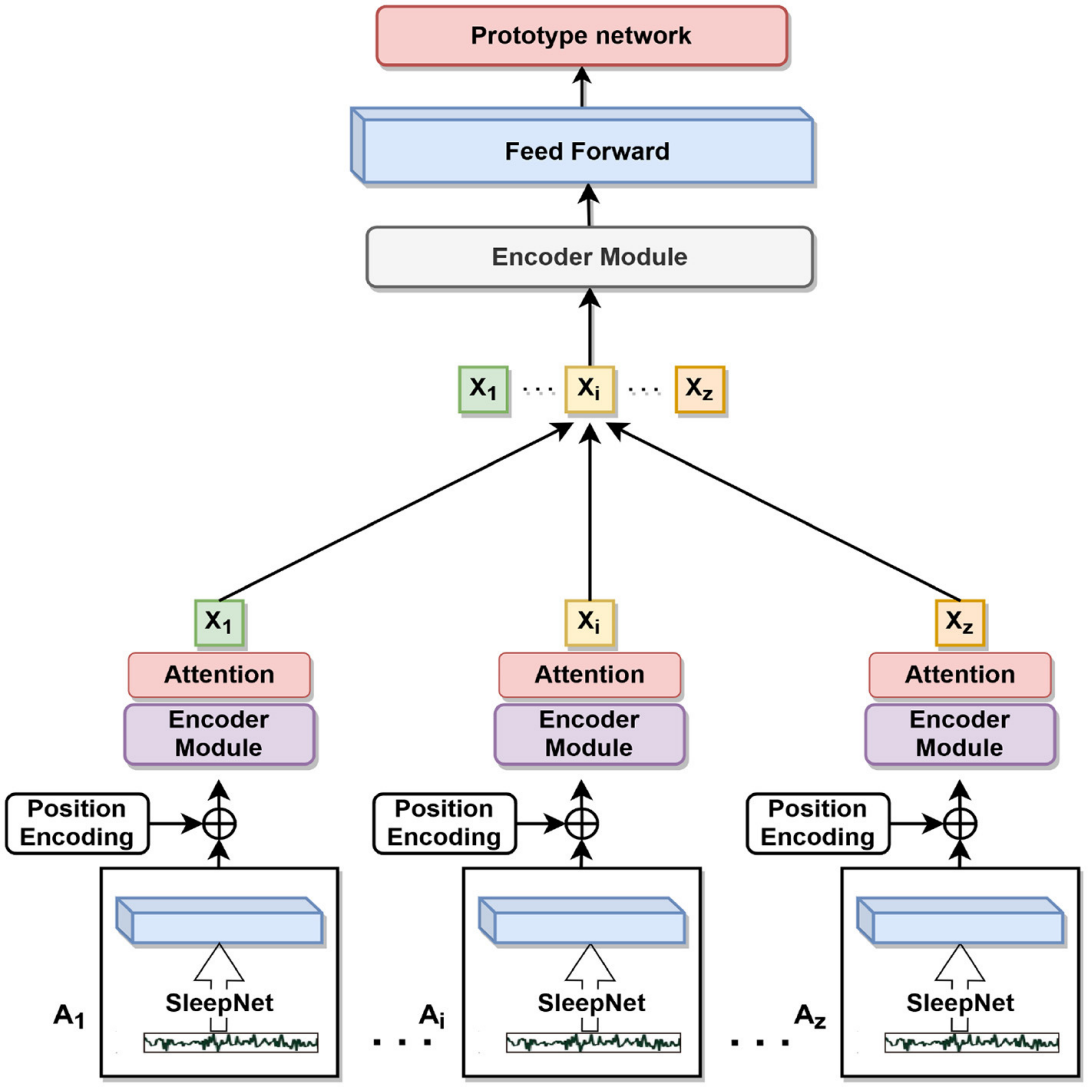
Backbone network.

In this study, we proposed a feature extraction network to analyze sleep EEG signals. The network consists of two main components: a convolutional neural network (SleepNet) and a multilayer transformer encoder module. Which is shown in Figure 4 and Stage B in Figure 1. The CNN is used to extract local features from the signals, while the transformer is used to capture global correlations between different parts of the signals.

#### 3.3.1 SleepNet features extraction

The CNN component comprises three convolutional layers with batch normalization and dropout, followed by a linear layer, as shown in the Table 1. The first layer has 64 filters with a kernel size of 64 and a stride of 16. The second layer has 128 filters with a kernel size of 8 and a pooling layer with a kernel size of 4. The third layer has 256 filters with a kernel size of 8 and a pooling layer with a kernel size of 4.

**Table 1:** CNN feature extraction.

**Layer**	**Kernel/filter**	**Stride/dilation**	**Output size (*p*)**
Input	–	–	1 × 3,000
Conv	64 × 1	Stride 16	64 × 187
BatchNorm	–	–	64 × 187
ReLU	–	–	64 × 187
Dropout	–	–	64 × 187
Conv	128 × 64	Dilation 1, Padding 1	128 × 184
BatchNorm	–	–	128 × 184
ReLU	–	–	128 × 184
MaxPool	4 × 1	Stride 4
Conv	256 × 128	Dilation 1, Padding 1	256 × 43
BatchNorm	–	–	256 × 43
ReLU	–	-	256 × 43
MaxPool	4 × 1	Stride 4	256 × 10
Dropout	–	–	256 × 10
Flatten	–	–	1 × 23,040
Linear	23,040 × 128	–	1 × 128

Formally, SleeNet extracts the ith feature from one EEG epoch *X*_i_, *CNN*_(_θ__*r*_)_ represents CNN converted from single channel EEG to eigenvector, and θ_*r*_ is the variable parameter of the CNN. The size of *f*_X_i__ depends on the sampling rate of input EEG. In the formula, *f* represents the CNN network we use. As shown in the following formula:


(12) $$\begin{array}{l} f_{\left(X_{i}\right)}={\text{CNN}}_{\left(\theta_{r}\right)}\left(X_{i}\right), \end{array}$$

the network is trained using the NAdam optimizer to minimize the cross-entropy loss.

#### 3.3.2 Transformer encoder module

The output of the third pooling layer is fed to the transformer component, which consists of encoder layers and transformer encoder. The encoder layer has a dimensionality of 128 and an attention mechanism. The encoder is applied to the input signals, and the output is averaged along the time axis before being fed to a fully connected layer with a 128-dimensional output. After the feedforward layer, our output feature vector is fed into the prototype network. After feature extraction of transformer encoder module, feature output formula is as follows:


(13) $$\begin{array}{l} F_{\left(X_{i}\right)}=\text{Encoder}\left(f\left(X_{i}\right)\right), \end{array}$$

where *F*_X_i__ represents features extracted by CNN and Transformer.

### 3.4 Model settings

Before using a prototypical network, we need to extract features from the collected data. We can construct a prototypical network architecture with five-way (1-shot, 3-shot, 5-shot, 10-shot, 15-shot, 20-shot, 25-shot). We randomly select 1, 3, 5, 10, 15, 20, and 25 epochs from the W, N1, N2, N3, and R phases of the meta-training set, respectively. The five sleep phases are preprocessed and SleepNet with transformer is used as our pre-trained neural network to compute prototypes for each sleep phase.

We divided a batch process into a support set and a query set, utilizing the embedding vectors of the support set to establish a class prototype. This prototype represented a typical embedding vector of a given class, and we then utilized values closely related to it for classification to compare the performance of our approach. In our experiment, Cosine distance, Manhattan distance, Euclidean distance, and Chebyshev distance were used as comparisons, and we obtain the Cosine distance as our best matching and most accurate measurement function. As such, we adopted the Cosine distance function as our distance evaluation metric.

To train the backbone network, we use the NAdam optimizer. To learn feature centers for distinct classes, we employ a stochastic gradient descent optimizer with a learning rate of 0.0009 and a center-loss weight of 0.0009. Among them, we use the pre-trained neural network to extract feature vectors, take the average value, conduct normalization processing, and use softmax for prediction analysis. Experiments were performed on 50 times and then fine-tuned by taking the average loss of gradient descent.

In the meta-testing, the remaining subjects from the meta-training were used as the meta-testing set, as a previously unseen category, for cross-subject EEG sleep staging. We randomly selected N samples in five sleep periods from the meta-testing set, and the five groups of samples were used as the meta-testing support set. Similar to meta-training, after building the initial prototype network on the first layer, we introduced the TDO method, including introducing a high confidence unlabeled query set as our support set, and recalculating the prototype network. All the rest sleep data is used as the meta-testing set, and the construction task is verified repeatedly. The average accuracy is taken as the accuracy of the final test result and the ACC, F1 values, and the accuracy of each category are obtained.

Since the experimental results may vary depending on the chosen support set sample, this experiment is repeated 50 times using the support set randomly, and the average precision is obtained as the final statistical result of the experiment. The average accuracy is taken as the accuracy of the final test result and the ACC, F1 values, and the accuracy of each category are obtained.

### 3.5 Evaluation

In our experiments, different subjects were used for cross-subject validation for meta-training and meta-testing subjects, and the meta-testing query set did not include meta-training subjects. Our experiments were conducted for 20 rounds, thus validating our experimental results. The experiment is a multi-class classification task for sleep staging. Accuracy, F-measure, recall, and kappa values are used to evaluate the performance of sleep staging. The overall performance is evaluated in terms of accuracy and Cohen's Kappa coefficient. The above evaluation metrics are formulated as follows:


(14) $$\begin{array}{l} Accuracy=\frac{TP+TN}{TP+FP+TN+FN}, \end{array}$$

(15) $$\begin{array}{l} \kappa =\frac{\text{Accuracy}-P_{e}}{1-P_{e}}, \end{array}$$

(16) $$\begin{array}{l} Recall=\frac{TP}{TP+FN}, \end{array}$$

(17) $$\begin{array}{l} Precision=\frac{TP}{TP+FP}, \end{array}$$

(18) $$\begin{array}{l} F_{1}=\frac{2\times \text{Recall}\times \text{Precision}}{\text{Precision}+\text{Recall}}, \end{array}$$

where TP is true positive, TN is true negative, FP is false positive, FN is false negative, and *P*_e_ is the hypothetical probability of chance agreement.

## 4 Results and discussion

In this section, we present a detailed analysis of the experimental results, including the performance of the sleep stage and the confusion matrix, and compare them with state-of-the-art experiments. We also conducted qualitative and quantitative experiments, including using different shots, different distance metric functions, and different learning rates. The t-SNE plots of the ablation experiments are also compared in the experiment to demonstrate the effectiveness of our experiment. We also discuss the feasibility of cross-channel sleep analysis and the limitations of our experiments.

### 4.1 Sleep stage scoring performance

In Table 2, we show the performance difference between the most advanced algorithm and our proposed prototypical network TPON on the Sleep-EDF-2013 dataset. It included two channels, Fpz-Cz and Pz-Oz (compared using five-way 15-shot method).

**Table 2:** The performance difference between the most advanced algorithm and our proposed TPON method.

**Database**	**System**	**Overall performance**			**Class-wise MF1**				
		**Acc**.	κ	**MF1**	**Wake**	**N1**	**N1**	**N3**	**REM**
SleepEDF-2013(Fpz-Cz)	MetaSleepLearner (Banluesombatkul et al., 2020)	72.1	0.62	64.8	70.0	29.0	78.6	79.1	67.5
	SleepEEGNet (Mousavi et al., 2019)	84.3	0.79	79.7	89.2	52.2	86.8	85.1	85.0
	DeepSleepNet (Supratak et al., 2017)	82.0	0.76	76.9	84.7	46.6	85.9	84.8	82.4
	DeepSleepNet-Lite (Fiorillo et al., 2021)	84.0	0.78	78.0	87.1	44.4	87.9	88.2	82.4
	AttnSleep (Eldele et al., 2021)	84.4	0.79	78.1	89.7	42.6	88.8	90.2	79.0
	SeqSleepNet+ (FT) (Phan et al., 2019)	85.2	0.79	79.6	89.2	52.2	86.8	85.1	85.0
	TinySleepNet (Supratak and Guo, 2020)	85.4	0.80	80.5	90.1	51.4	88.5	88.3	84.3
	XSleepNet2 (Phan et al., 2021)	86.3	0.81	80.6	**92.2**	51.8	88.0	86.8	83.9
	XSleepNet1 (Phan et al., 2021)	86.0	0.81	80.0	91.3	49.5	88.0	86.9	84.2
	MNN (Jiang et al., 2020)	85.9	−	80.5	84.6	56.3	90.7	84.8	86.1
	Khalili & Asl (Khalili and Asl, 2021)	85.4	0.80	79.3	90.0	46.6	88.4	86.1	84.6
	U-Sleep (Perslev et al., 2021)	−	−	79.0	93.0	**57.0**	86.0	71.0	**88.0**
	SleepFCN (Goshtasbi et al., 2022)	84.8	0.78	78.8	89.6	44.6	89.1	90.6	80.3
	ResNetMHA (Qu et al., 2020)	84.3	−	79.0	90.2	48.3	87.8	85.6	83.3
	IITNet (Seo et al., 2020)	83.9	0.78	77.6	−	−	−	−	−
	FCNN + RNN (Phan et al., 2021)	81.8	0.75	75.6	89.4	44.1	84.0	84.0	76.3
	*TPON (Ours)*	**87.1**	**0.82**	**81.7**	91.9	50.9	**91.1**	**91.9**	82.5
SleepEDF-2013(Pz-Oz)	DeepSleepNet (Supratak et al., 2017)	79.8	0.72	73.1	88.1	37	82.7	77.3	80.3
	SleepEEGNet (Mousavi et al., 2019)	82.8	0.77	**77.0**	90.3	**44.6**	85.7	81.6	**82.9**
	*TPON (Ours)*	**83.3**	**0.77**	73.5	**90.8**	23.8	**88.2**	**86.4**	78.3

The bold values represent the metrics in the table that achieved the best result in a particular method.

Our proposed meta learning cross-subject sleep segmentation algorithm TPON can be seen from Tables 2, 3. Under the Fpz-Cz channel, we use fewer subjects and samples than other meta-learning algorithms, such as MetaSleepLearner. However, the accuracy is improved by 15%, and the F1 score and MF1 for the five sleep epochs are higher than those of MAML's MetaSleepLearner using Meta-learning, achieving a phased achievement. Compared to the traditional deep learning algorithm DeepSleepNet, the overall accuracy of TPON under the Fpz-Cz channel is also improved by 5.1%. This is a pioneering use of meta-learning algorithms and makes them comparable in accuracy to traditional machine learning algorithms. Cohen's Kappa values increased to 0.82 and MF1 score increased to 81.7. In the Pz-Oz channel, the accuracy rate reached 83.3, the MF1 reached 73.5, and Cohen's Kappa value reached 0.77.

**Table 3:** PR, RE, and F1 performance in Fpz-Cz and Pz-Oz channels from the SleepEDF-2013 datase.

	**Fpz-Cz**			**Pz-Oz**		
**Stages**	**PR**	**RE**	**F1**	**PR**	**RE**	**F1**
Wake	91.4	92.3	91.9	87.9	93.9	90.8
N1	53.7	48.3	50.9	38.6	17.2	23.8
N2	94.1	88.3	91.1	90.6	85.8	88.2
N3	89.1	94.8	91.9	81.9	91.5	86.4
REM	78.1	87.5	82.5	72.7	84.8	78.3

TPON is a prototype network algorithm proposed by us. It can better train our sleep EEG, after extracting sleep features from the backbone network. TPON can be effectively mapped into the space to achieve a better classification effect. In the process of cross-subject identification, we can also train and identify our unfamiliar subjects across the subject to achieve a more accurate classification effect.

To perform a comprehensive experimental analysis of the proposed TPON, we performed ablation experiments, as can be seen in Table 4. Our ablation experiments are compared with our TPON using the full experimental procedure. The first method is TPON. The second approach is to remove the attention mechanism. The third approach excludes the TDO mechanism. Finally, the fourth approach excludes the attention mechanism and the TDO mechanism. The fifth method excludes the TDO mechanism and the prototype network and adopts the traditional deep learning approach. The results of the ablation experiments are shown in Table 4. From Table 4, it can be seen that TPON is the best sleep staging method in our ablation experiments.

**Table 4:** Ablation study on TPON.

**Channel**	**Attention**	**TDO**	**Prototype**	**Class-wise MF1**					**Performance**		
				**Wake**	**N1**	**N2**	**N3**	**REM**	**Acc**.	κ	**MF1**
***Fpz-Cz***	✓	✓	✓	91.9	**50.9**	91.1	**91.9**	**82.5**	**87.1**	**0.82**	**81.7**
***Fpz-Cz***	×	✓	✓	90.8	36.4	91.1	85.9	81.0	86.6	0.81	77.0
***Fpz-Cz***	✓	×	✓	**92.0**	39.0	**91.5**	90.9	81.2	86.6	0.82	78.9
***Fpz-Cz***	×	×	✓	92.0	39.8	91.3	89.2	80.9	86.4	0.80	78.6
***Fpz-Cz***	✓	×	×	91.2	41.7	88.7	90.7	81.0	85.1	0.80	78.7
***Pz-Oz***	✓	✓	✓	**90.8**	23.8	**88.2**	86.4	78.3	**83.3**	**0.77**	**73.5**
***Pz-Oz***	×	✓	✓	90.4	23.7	88.0	86.2	78.2	83.0	0.76	73.3
***Pz-Oz***	✓	×	✓	89.7	**26.1**	86.5	86.5	78.9	82.8	0.77	73.5
***Pz-Oz***	×	×	✓	90.0	24.7	88.1	**86.5**	78.0	82.9	0.74	73.5
***Pz-Oz***	✓	×	×	88.7	25.7	87.3	85.9	**79.2**	82.1	0.73	73.4

Our ablation experiments are compared with our TPON using the full experimental procedure. The first method is TPON. The second approach is to remove the attention mechanism. The third approach excludes the TDO mechanism. The fourth approach excludes the attention mechanism and the TDO mechanism. The fifth method excludes the TDO mechanism and the prototype network and adopts the traditional deep learning approach. Our ablation experiments were conducted on two channels, Fpz-Cz and Pz-Oz. The bold values represent the metrics in the table that achieved the best result in a particular method.

**Figure 5:**
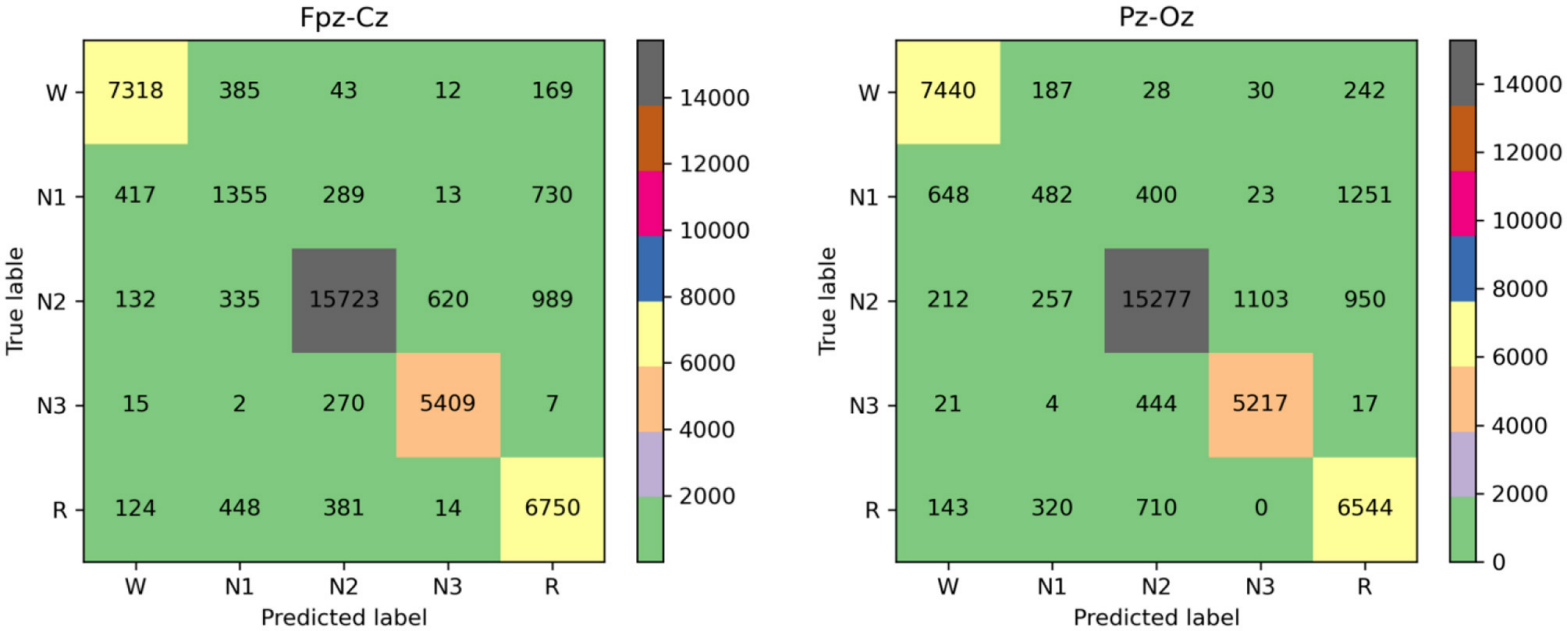
Confusion matrix obtained Fpz-Cz and Pz-Oz channels from the SleepEDF-2013 dataset.

Figure 5 shows confusion matrices on the Fpz-Cz and Pz-Oz channels from the Sleep-EDF datasets. Each row and column represent the number of 30-s EEG epochs of each sleep stage classifified by the sleep expert and our model, respectively. The last three columns in each row indicate per-class performance metrics computed from the confusion matrix. Table 3 shows the PR, RE, and F1 performance in Fpz-Cz and Pz-Oz channels from the SleepEDF-2013 datase. PR means precision, RE means recall, and F1 means F-measure.

### 4.2 Training time performance

In this section, we will calculate the training time of our proposed few-shot EEG sleep staging algorithm, TPON, based on the prototype network. This includes the training time for each validation fold on each node, with a total of 20 validation folds.

Our proposed few-shot EEG sleep classification algorithm effectively solves the problem of long training time in traditional deep learning. The network initialization can quickly adapt to new tasks and has the ability to train models on a small number of samples, including “learning to learn” features. TPON introduces a prototype-based meta-learning algorithm during the training process, greatly reducing the time for single test validation. The training time for each validation fold is ~22 min, which greatly saves computation energy and reduces the time for doctors to manually stage patients.

### 4.3 Consideration of N-shot learning

**Figure 6:**
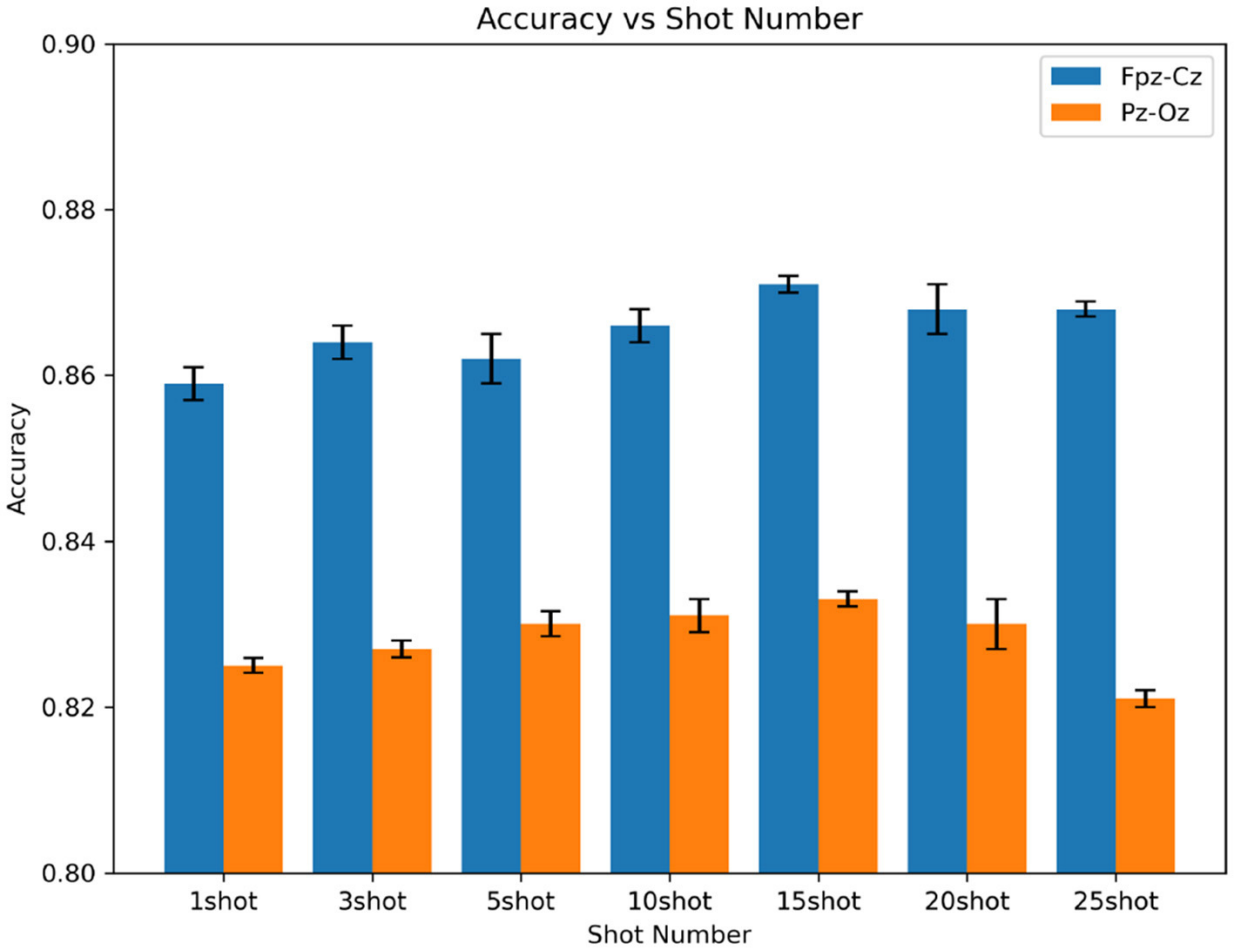
The accuracy of different shot quantities in the Fpz-Cz and Pz-Oz channels.

In five-way N-shot, we analyze the effect of variation in the number of shot samples on the accuracy of sleep quintet classification and MF1 measurement. We show the accuracy of different shot quantities in the Fpz-Cz and Pz-Oz channels, which is shown in Figure 6. It can be seen that the accuracy of the experiment increases with the number of shots. In the Fpz-Cz channel, the highest accuracy is 87.1% at 10-shot. In the Pz-Oz channel, the highest accuracy is 83.3% at 15-shot.

We can see that the five-way 15-shot method of using few-shot learning is similar to the traditional method of using deep learning with a large number of data samples.

### 4.4 Different metric distance functions

**Figure 7:**
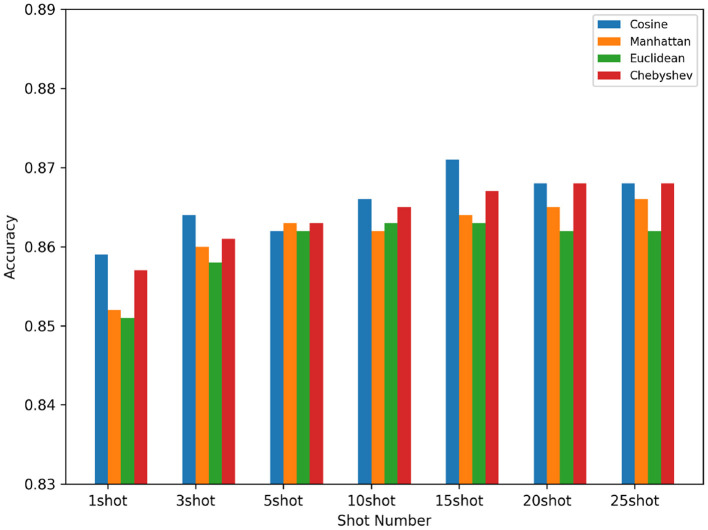
Compare different distance measurement functions in different shot.

We compare different distance measurements functions in Fpz-Cz channel which is shown in Figure 7. In the prototypical networks, we use different distance metric functions as our benchmarks, and finally obtain that the most efficient distance metric function is the Cosine distance function. In our experiments, the Cosine distance, the Manhattan distance, the Euclidean distance, and the Chebyshev distance are used as comparisons. We can see from the following figure that under the condition of using the same five-way (1-shot to 25-shot), and using different distance measurement functions, the prototypical networks has different effects. Finally, we choose the best performing Cosine distance as the metric function for our prototypical network.

### 4.5 Performance of t-SNE visualization in the attention mechanism ablation experiment

**Figure 8:**
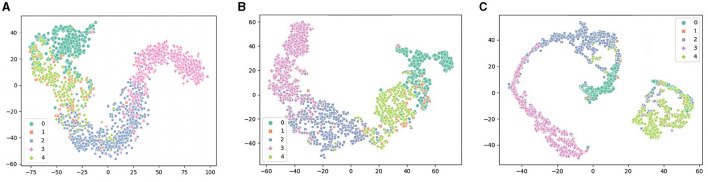
Performance of t-SNE visualization in the TDO and attention mechanism ablation experiment. **(A)** Remove the TDO mechanism. **(B)** Remove the attention mechanism. **(C)** Transductive prototype optimization network (TPON).

In this section, we will present three t-SNE plots, which are shown in Figure 8. We used ablation experiments to compare TPON performance without a TDO, attentional mechanism. By comparing the t-SNE plots of the two cases, we can observe that the use of the TDO and attention mechanism leads to a clear clustering effect, a more reasonable sample distribution, and a better representation of the distance between samples of different classes. This indicates that we are able to capture the differences between different classes more accurately with the TDO and attention mechanism. Therefore, we can conclude that in sleep EEG staging, the use of TDO and attention mechanisms can increase the performance of classification.

### 4.6 The effect of learning rate

**Figure 9:**
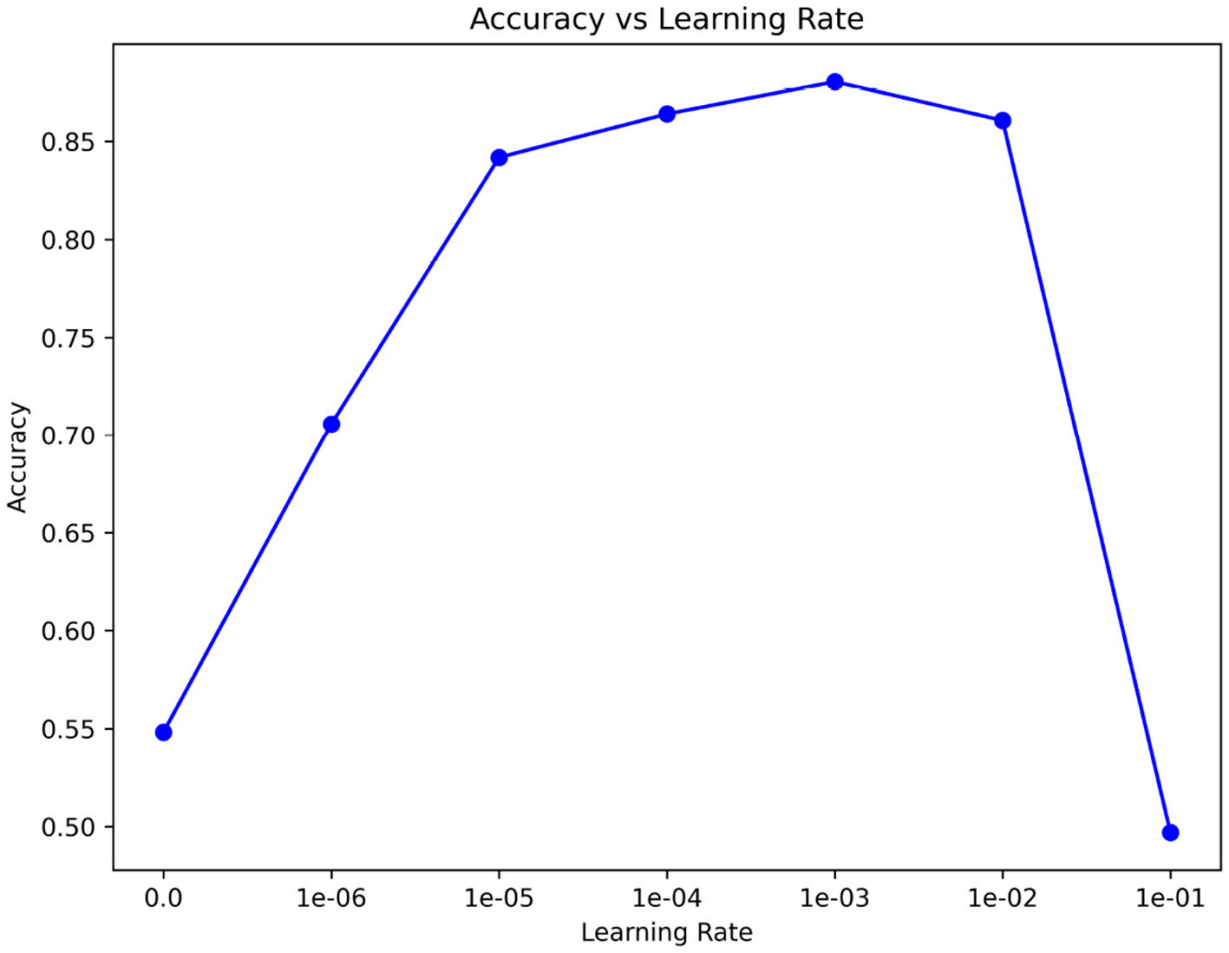
The effect of learning rate.

In Figure 9, we consider the impact of learning rate on our backbone network feature extraction. Therefore, we use a variety of ways to compare learning rates. We use the 15th subject as our meta-testing subject to reduce training time. In the meta-training phase and meta-testing phase, we used to select the most suitable learning rate η∈{1 × 10^−1^, 1 × 10^−2^, 1 × 10^−3^, 1 × 10^−4^, 1 × 10^−5^, 1 × 10^−6^, 0}, as well as the number of training iterations. The default maximum training iterations were set to 50, respectively. It can be seen that there are significant differences in the feature extraction effect among different learning rates. When the learning rate is 1 × 10^−3^, the learning effect is the best. After subdivision learning, we finally determined the learning rate to be 0.0009.

### 4.7 Cross-channel sleep staging

In the meta-learning, the meta-training stage trains the ability of the model to “learn to learn.” We propose the hypothesis that the manually segmented sleep data by the physician has only one channel. We need to classify the sleep EEG signals of another channel. So, we can perform meta training on the sleep data of existing channels. Then, the model not only has the ability to “learn to learn” but also has the ability to recognize unfamiliar channel data to a certain extent.

Therefore, we propose a cross-channel EEG recognition network. The key to this idea is to use and train on EEG sleep data from known channels, and then perform sleep staging on EEG sleep data from unfamiliar channels. Our experiment uses the same mechanism as TPON. The only difference is that our meta-training subjects and meta-testing subjects used different sleep channels. Adopting this approach is to simulate the real-life situation described above.

Our experiment used Pz-Oz channel data as the meta-training set and Fpz-Oz channel data as the meta-testing set. The data of the Pz-Oz channel include 19 subjects, while the data of the Fpz-Oz channel uses the remaining subjects. To ensure the rigor of the experiment, we repeated it 20 times and calculated the average value.

From the experimental results, our cross-channel EEG sleep staging achieves good results. Especially in the case of five-way 25-shot, the accuracy is 82.3%, which has reached a high level.

### 4.8 Limitations of the study

Our dataset refers to the dataset adopted by DeepSleepNet, using the data of 20 SC subjects (healthy subjects) in Sleep-EDF, which included 20 healthy subjects (26 to 35 years old), including 10 healthy men and 10 healthy women. But we might be dealing with real-world people with sleep disorders, so the results could be biased.

There is another limitation here, which is that performance is slightly worse when there is a large difference between labeled and unlabeled data. This includes training with one type of data and testing with another, and the data are very different, which may be slightly less effective in our cross-data testing.

In the t-SNE diagram of TPON (Stage A and Stage B in Figure 8) we learned, we can see that our W, N2, N3, and R stages have obvious distribution intervals, and the distribution differences are obvious. However, the N1 phase is not better separated and mixed with the R phase, which causes difficulties in our segmentation, and many N1 phases are misclassified as R phases.

## 5 Conclusion

In this study, we propose a few-shot EEG sleep staging based on transductive prototype optimization network (TPON) method. A modified version of the prototypical network algorithm was used for the experiments, and the Cosine distance function was used as the distance metric function. Given the diverse nature of EEG sleep data across subjects, efficient adaptation and training with new data from previously unseen subjects remains a significant challenge. Our future work is to experimentally improve the problem of having too few N1 stages in the meta-testing dataset. The low accuracy for N1 staging can be explained by the fact that most of the disagreements occurred during transitions between sleep stages and N1 stage typically has a lower bout length (number of consecutive 30-s epochs scored as N1) compared to the other stages (Rosenberg and Van Hout, 2014). Although the problem of having too few N1 stages is related to the proportion of N1 stages in the whole night during human sleep, we can introduce a relevant proportionality coefficient to solve the problem of having too low a fraction of N1. Our future research directions also include the adoption of more advanced meta-learning algorithms, followed by the improvement of our backbone network and the adoption of dynamic convolutional neural networks to address the problem of imbalanced sample distributions and too few support set samples in few-shot learning.

## Data availability statement

Publicly available datasets were analyzed in this study. This data can be found at: https://physionet.org/content/sleep-edfx/1.0.0/.

## Ethics statement

Ethical approval was not required for the study involving humans in accordance with the local legislation and institutional requirements. Written informed consent to participate in this study was not required from the participants or the participants' legal guardians/next of kin in accordance with the national legislation and the institutional requirements.

## Author contributions

JL: Formal analysis, Investigation, Methodology, Writing—original draft. CW: Formal analysis, Investigation, Methodology, Software, Writing—original draft. JP: Conceptualization, Project administration, Validation, Writing—review & editing. FW: Methodology, Supervision, Validation, Writing—review & editing.

## References

[B1] Allison B. Z. Wolpaw E. W. Wolpaw J. R. (2007). Brain-computer interface systems: progress and prospects. Expert Rev. Med. Devices 4, 463–474. 10.1586/17434440.4.4.463 17605682

[B2] Alzubaidi L. Zhang J. Humaidi A. J. Al-Dujaili A. Duan Y. Al-Shamma O.. (2021). Review of deep learning: concepts, cnn architectures, challenges, applications, future directions. J. Big Data 8, 1–74. 10.1186/s40537-021-00444-8 33816053 PMC8010506

[B3] Amin S. U. Alsulaiman M. Muhammad G. Mekhtiche M. A. Hossain M. S. (2019). Deep learning for EEG motor imagery classification based on multi-layer cnns feature fusion. Future Gener. Comput. Syst. 101, 542–554. 10.1016/j.future.2019.06.027

[B4] Andreotti F. Phan H. Cooray N. Lo C. Hu M. T. De Vos M. (2018). ‘Multichannel sleep stage classification and transfer learning using convolutional neural networks,” in 2018 40th Annual International Conference of the IEEE Engineering in Medicine and Biology Society (EMBC) (Honolulu, HI: IEEE), 171–174. 10.1109/EMBC.2018.8512214 30440365

[B5] Arel I. Rose D. C. Karnowski T. P. (2010). Deep machine learning-a new frontier in artificial intelligence research [research frontier]. IEEE Comput. Intell. Mag. 5, 13–18. 10.1109/MCI.2010.938364

[B6] Arrieta A. B. Díaz-Rodríguez N. Del Ser J. Bennetot A. Tabik S. Barbado A.. (2020). Explainable artificial intelligence (XAI): Concepts, taxonomies, opportunities and challenges toward responsible AI. Inf. Fusion 58, 82–115. 10.1016/j.inffus.2019.12.012

[B7] Banluesombatkul N. Ouppaphan P. Leelaarporn P. Lakhan P. Chaitusaney B. Jaimchariyatam N.. (2020). Metasleeplearner: a pilot study on fast adaptation of bio-signals-based sleep stage classifier to new individual subject using meta-learning. IEEE J. Biomed. Health Inf. 25, 1949–1963. 10.1109/JBHI.2020.3037693 33180737

[B8] Berry R. B. Brooks R. Gamaldo C. E. Harding S. M. Marcus C. Vaughn B. V.. (2012). The AASM Manual for the Scoring of Sleep and Associated Events: Rules, Terminology and Technical Specifications. Darien, IL: American Academy of Sleep Medicine, 176.

[B9] Boostani R. Karimzadeh F. Nami M. (2017). A comparative review on sleep stage classification methods in patients and healthy individuals. Comput. Methods Programs Biomed. 140, 77–91. 10.1016/j.cmpb.2016.12.004 28254093

[B10] Carskadon M. A. Dement W. C. Kryger M. H. Roth T. Roehrs T. A. (2005). “Normal human sleep: an overview,” in Principles and Practice of Sleep Medicine, 4th ed., eds M. H. Kryger, T. Roth, and W. C. Dement (Philadelphia, PA: Elsevier Saunders), 13–23. 10.1016/B0-72-160797-7/50009-4

[B11] Chambon S. Galtier M. N. Arnal P. J. Wainrib G. Gramfort A. (2018). A deep learning architecture for temporal sleep stage classification using multivariate and multimodal time series. IEEE Trans. Neural Syst. Rehabil. Eng. 26, 758–769. 10.1109/TNSRE.2018.2813138 29641380

[B12] Chen S. Ma B. Zhang K. (2009). On the similarity metric and the distance metric. Theor. Comput. Sci. 410, 2365–2376. 10.1016/j.tcs.2009.02.023

[B13] Dietterich T. G. (1997). Machine-learning research. AI Mag. 18, 97–97.

[B14] Dong H. Supratak A. Pan W. Wu C. Matthews P. M. Guo Y.. (2017). Mixed neural network approach for temporal sleep stage classification. IEEE Trans. Neural Syst. Rehabil. Eng. 26, 324–333. 10.1109/TNSRE.2017.2733220 28767373

[B15] Eldele E. Chen Z. Liu C. Wu M. Kwoh C.-K. Li X.. (2021). An attention-based deep learning approach for sleep stage classification with single-channel EEG. IEEE Trans. Neural Syst. Rehabil. Eng. 29, 809–818. 10.1109/TNSRE.2021.3076234 33909566

[B16] Finn C. Abbeel P. Levine S. (2017). “Model-agnostic meta-learning for fast adaptation of deep networks,” in International Conference on Machine Learning (Sydney, NSW: PMLR), 1126–1135.

[B17] Fiorillo L. Favaro P. Faraci F. D. (2021). Deepsleepnet-lite: a simplified automatic sleep stage scoring model with uncertainty estimates. IEEE Trans. Neural Syst. Rehabil. Eng. 29, 2076–2085. 10.1109/TNSRE.2021.3117970 34648450

[B18] Galán F. Nuttin M. Lew E. Ferrez P. W. Vanacker G. Philips J.. (2008). A brain-actuated wheelchair: asynchronous and non-invasive brain-computer interfaces for continuous control of robots. Clin. Neurophysiol. 119, 2159–2169. 10.1016/j.clinph.2008.06.001 18621580

[B19] Goshtasbi N. Boostani R. Sanei S. (2022). Sleepfcn: a fully convolutional deep learning framework for sleep stage classification using single-channel electroencephalograms. IEEE Trans. Neural Syst. Rehabil. Eng. 30, 2088–2096. 10.1109/TNSRE.2022.3192988 35862320

[B20] Hori T. Sugita Y. Koga E. Shirakawa S. Inoue K. Uchida S.. (2001). Proposed supplements and amendments to ‘a manual of standardized terminology, techniques and scoring system for sleep stages of human subjects', the rechtschaffen and kales (1968) standard. Psychiatry Clin. Neurosci. 55, 305–310. 10.1046/j.1440-1819.2001.00810.x 11422885

[B21] Hramov A. E. Maksimenko V. A. Pisarchik A. N. (2021). Physical principles of brain-computer interfaces and their applications for rehabilitation, robotics and control of human brain states. Phys. Rep. 918, 1–133. 10.1016/j.physrep.2021.03.002

[B22] Ismail W. W. Hanif M. Mohamed S. Hamzah N. Rizman Z. I. (2016). Human emotion detection via brain waves study by using electroencephalogram (EEG). Int. J. Adv. Sci. Eng. Inf. Technol. 6, 1005–1011. 10.18517/ijaseit.6.6.1072

[B23] Jiang X. Wang H. Chen Y. Wu Z. Wang L. Zou B.. (2020). MNN: a universal and efficient inference engine. Proc. Mach. Learn. Syst. 2, 1–13. 10.48550/arXiv.2002.12418

[B24] Khalili E. Asl B. M. (2021). Automatic sleep stage classification using temporal convolutional neural network and new data augmentation technique from raw single-channel EEG. Comput. Methods Programs Biomed. 204, 106063. 10.1016/j.cmpb.2021.106063 33823315

[B25] Korkalainen H. Aakko J. Nikkonen S. Kainulainen S. Leino A. Duce B.. (2019). Accurate deep learning-based sleep staging in a clinical population with suspected obstructive sleep apnea. IEEE J. Biomed. Health Inf. 24, 2073–2081. 10.1109/JBHI.2019.2951346 31869808

[B26] LeCun Y. Bengio Y. Hinton G. (2015). Deep learning. Nature 521, 436–444. 10.1038/nature14539 26017442

[B27] Liu X. Liu L. Liu H. Zhang X. (2023). Capturing the few-shot class distribution: Transductive distribution optimization. Pattern Recognit. 138, 109371. 10.1016/j.patcog.2023.109371

[B28] Mousavi S. Afghah F. Acharya U. R. (2019). Sleep EEG net: automated sleep stage scoring with sequence to sequence deep learning approach. PLoS ONE 14, e0216456. 10.1371/journal.pone.0216456 31063501 PMC6504038

[B29] Perslev M. Darkner S. Kempfner L. Nikolic M. Jennum P. J. Igel C.. (2021). U-sleep: resilient high-frequency sleep staging. NPJ Digit. Med. 4, 72. 10.1038/s41746-021-00440-5 33859353 PMC8050216

[B30] Perslev M. Jensen M. Darkner S. Jennum P. J. Igel C. (2019). U-time: a fully convolutional network for time series segmentation applied to sleep staging. Adv. Neural Inf. Process. Syst. 32. 10.48550/arXiv.1910.11162

[B31] Phan H. Andreotti F. Cooray N. Chén O. Y. De Vos M. (2018). Joint classification and prediction cnn framework for automatic sleep stage classification. IEEE Trans. Biomed. Eng. 66, 1285–1296. 10.1109/TBME.2018.2872652 30346277 PMC6487915

[B32] Phan H. Andreotti F. Cooray N. Chén O. Y. De Vos M. (2019). Seqsleepnet: end-to-end hierarchical recurrent neural network for sequence-to-sequence automatic sleep staging. IEEE Trans. Neural Syst. Rehabil. Eng. 27, 400–410. 10.1109/TNSRE.2019.2896659 30716040 PMC6481557

[B33] Phan H. Chén O. Y. Tran M. C. Koch P. Mertins A. De Vos M. (2021). Xsleepnet: multi-view sequential model for automatic sleep staging. IEEE Trans. Pattern Anal. Mach. Intell. 44, 5903–5915. 10.1109/TPAMI.2021.3070057 33788679

[B34] Qu W. Wang Z. Hong H. Chi Z. Feng D. D. Grunstein R.. (2020). A residual based attention model for EEG based sleep staging. IEEE J. Biomed. Health Inf. 24, 2833–2843. 10.1109/JBHI.2020.2978004 32149700

[B35] Rosenberg R. S. Van Hout S. (2014). The american academy of sleep medicine inter-scorer reliability program: respiratory events. J. Clin. Sleep Med. 10, 447–454. 10.5664/jcsm.3630 24733993 PMC3960390

[B36] Sadeh A. (2015). III sleep assessment methods. Monogr. Soc. Res. Child Dev. 80, 33–48. 10.1111/mono.12143 25704734

[B37] Samy L. Huang M.-C. Liu J. J. Xu W. Sarrafzadeh M. (2013). Unobtrusive sleep stage identification using a pressure-sensitive bed sheet. IEEE Sens. J. 14, 2092–2101. 10.1109/JSEN.2013.2293917

[B38] Schultz M. Joachims T. (2003). Learning a distance metric from relative comparisons. Adv. Neural Inf. Process. Syst. 16, 41–48.

[B39] Seo H. Back S. Lee S. Park D. Kim T. Lee K.. (2020). Intra-and inter-epoch temporal context network (IITNET) using sub-epoch features for automatic sleep scoring on raw single-channel EEG. Biomed. Signal Process. Control 61, 102037. 10.1016/j.bspc.2020.102037

[B40] Shi W.-J. Yu J. Zhu X.-J. Lin Y.-F. (2023). Meta transfer learning sleep stage classification model in few-shot scenarios. J. Comput. Appl. 10.11772/j.issn.1001-9081.2023050747

[B41] Snell J. Swersky K. Zemel R. (2017). Prototypical networks for few-shot learning. Adv. Neural Inf. Process. Syst. 30, 4080–4090.

[B42] Sun C. Chen C. Li W. Fan J. Chen W. (2019). A hierarchical neural network for sleep stage classification based on comprehensive feature learning and multi-flow sequence learning. IEEE J. Biomed. Health Inf. 24, 1351–1366. 10.1109/JBHI.2019.2937558 31478877

[B43] Sung F. Yang Y. Zhang L. Xiang T. Torr P. H. Hospedales T. M.. (2018). “Learning to compare: Relation network for few-shot learning,” in Proceedings of the IEEE Conference on Computer Vision and Pattern Recognition (Salt Lake City, UT: IEEE), 1199–1208. 10.1109/CVPR.2018.00131

[B44] Supratak A. Dong H. Wu C. Guo Y. (2017). Deepsleepnet: a model for automatic sleep stage scoring based on raw single-channel EEG. IEEE Trans. Neural Syst. Rehabil. Eng. 25, 1998–2008. 10.1109/TNSRE.2017.2721116 28678710

[B45] Supratak A. Guo Y. (2020). “Tinysleepnet: an efficient deep learning model for sleep stage scoring based on raw single-channel EEG,” in 2020 42nd Annual International Conference of the IEEE Engineering in Medicine and Biology Society (EMBC) (Montreal, QC: IEEE), 641–644. 10.1109/EMBC44109.2020.9176741 33018069

[B46] Tsinalis O. Matthews P. M. Guo Y. (2016). Automatic sleep stage scoring using time-frequency analysis and stacked sparse autoencoders. Ann. Biomed. Eng. 44, 1587–1597. 10.1007/s10439-015-1444-y 26464268 PMC4837220

[B47] Vanschoren J. (2019). “Meta-learning,” in Automated Machine Learning: Methods, Systems, Challenges, eds F. Hutter, L. Kotthoff, and J. Vanschoren (Cham: Springer), 35–61. 10.1007/978-3-030-05318-5_2

[B48] Wang Y. Yao Q. Kwok J. T. Ni L. M. (2020). Generalizing from a few examples: a survey on few-shot learning. ACM Comput. Surv. 53, 1–34. 10.1145/3386252

[B49] Zhu Y. Liu C. Jiang S. (2020). “Multi-attention meta learning for few-shot fine-grained image recognition,” in IJCAI (Beijing), 1090–1096. 10.24963/ijcai.2020/152

